# Comparative metagenomic analysis of human intervertebral disc nucleus pulposus and cartilaginous end plates

**DOI:** 10.3389/fcvm.2022.927652

**Published:** 2022-09-28

**Authors:** Rajasekaran Shanmuganathan, Chitraa Tangavel, Sri Vijay Anand K S, Raveendran Muthurajan, Sharon Miracle Nayagam, Monica Steffi Matchado, Sunmathi Rajendran, Rishi Mugesh Kanna, Ajoy Prasad Shetty

**Affiliations:** ^1^Department of Spine Surgery, Ganga Hospital, Coimbatore, India; ^2^Department of Biotechnology, Ganga Research Centre, Coimbatore, India; ^3^Department of Plant Biotechnology, Tamil Nadu Agricultural University, Coimbatore, India

**Keywords:** intervertebral disc, disc degeneration, bacteria, microbiome, endplate, dysbiosis, next-generation sequencing

## Abstract

**Study design:**

The diversity of microflora inhabiting endplate (EP) and nucleus pulposus (NP) tissues of human intervertebral disc (IVD) was profiled through NGS-supported 16S rRNA amplicon sequencing. Sixteen EP and their corresponding NP were excised from the brain-dead voluntary organ donors with no clinical history of low back pain, and 12 herniated and 8 degenerated NP tissues isolated from the patients undergoing spinal surgery were subjected to study the alteration in the microbial diversity.

**Objective(s):**

To understand in normal IVD, whether the colonization of bacteria to the NP is through the EP in discs with intact annulus fibrosus. To identify significantly differing microbial population(s) between normal and diseased IVD (NP).

**Background of the study:**

There is increasing evidence for subclinical infection by fastidious low, growing bacteria to be a cause of disc degeneration. Although the presence of bacteria in NP has been reported well in literature, the source of bacteria is not clearly proved as the disc is avascular in healthy condition. Documentation of similar bacterial populations in the EP and NP may add proof that bacterial inoculation of NP occurs *via* the EP.

**Materials and methods:**

Sixteen EP and their corresponding NP excised from brain-dead voluntary organ donors with no history of back pain and 20 diseased discs collected from patients undergoing microdiscectomy/fusion surgery were used for profiling microbiome through 16S rRNA amplicon sequencing using primers specific for V1-V9 hypervariable regions. Changes in bacterial diversity and abundance were analysed to identify the key microbial populations in normal IVD NP and EP tissues and those significantly altered in diseased IVD (NP).

**Results:**

NP and EP shared a similar spectrum of microbiome but with varying abundance. The five dominant phyla identified were *Proteobacteria, Firmicutes, Actinobacteria, OD1, and Bacteroidetes. Proteobacteria* was found to be the most abundant phyla in both NP (62%) and EP (53%) of the normal IVD. This was followed by *Firmicutes (16%), Actinobacteriota (11%), OD1 (Parcubacteria) (7.6%), and Bacteroidetes (2%) in NP and Firmicutes (23.4%), OD1 (Parcubacteria) (17.6%), Actinobacteriota (2.8%), and Bacteroidetes (2.6%) in EP, respectively*. Under diseased conditions, *Proteobacteria* (68%) was dominant when compared with other phyla. However, there was no significant difference in the abundance of *Proteobacteria* between the normal and diseased discs. Interestingly, the other dominant phyla such as *Firmicutes* (Normal-NP: 16.2%; Diseased-NP: 4.02%) and *Actinobacteria* (Normal-NP: 11%; Diseased-NP: 0.99%) showed a significant reduction in degenerated discs. To understand the key microbial populations that are significantly altered during disease, correlation analysis was performed among the three phyla, which revealed a negative correlation in the ratio of *Actinobacteria* + *Firmicutes vs. Proteobacteria* (*p* = 0.001) in DD.

**Conclusion:**

Results of our study clearly demonstrated a similar bacterial diversity but with varying abundance between the EP and NP, suggesting the existence of the endplate–nucleus pulposus axis in the normal IVD microbiome. Further, our results have indicated that the changes in the abundance of *Actinobacteria* + *Firmicutes vs. Proteobacteria* during DDD need further investigation.

## Introduction

Low back pain (LBP) due to degenerative disc disease (DDD) is fast becoming the most common cause of musculoskeletal disability, resulting in a huge socioeconomic burden across all nations (1). The prevalence of LBP has increased from 377.5 million in 1990 to 577.0 million in 2017, and greater attention is needed to mitigate the global burden of LBP (2). While mechanical, traumatic, genetic susceptibility, smoking, inflammaging, physiological, and work profile have been postulated as various causes of DDD and LBP, there is mounting evidence for microbial etiology as well (3).

In our previous study, we documented for the first time that human lumbar intervertebral discs (IVD), even in normal asymptomatic subjects, were not sterile and harbored a distinct microbiome (4). While the human microbiome has a crucial role in supporting and maintaining health, alteration in relative abundance or diversity (dysbiosis) of microflora causes changes in the accumulation of microbial proteins, peptides, and metabolites, disturbing the intense crosstalk mechanisms between the host and microbiome, affecting health. Dysbiosis in both the gut and skin has clearly been linked to many disorders, such as inflammatory bowel diseases (IBD), irritable bowel syndrome (IBS), diabetes, obesity, cancer, and cardiovascular and central nervous system disorders (5). More recently, the gut microbiota has been associated with chronic musculoskeletal pain syndromes, including LBP and fibromyalgia (6, 7).

The disc being an avascular structure, the source of bacteria is unclear when the annulus fibrosis is still intact (8). In the current study, we investigate for the first time the endplate–nucleus pulposus axis in the normal human IVD microbiome. We further analyzed the microbial diversity of IVD between health and disease conditions which has thrown further insights into the discovery of metagenomic biomarkers for the diagnosis of IVD disease.

## Materials and methods

### Sample population

Lumbar discs from a brain-dead voluntary organ donor(s) and patients undergoing microdiscectomy/spinal fusion were obtained. The normal discs (ND) group had 16 NP (Nucleus Pulposus) tissues and 16 EP (Cartilaginous Endplates) tissues excised from MRI normal discs of brain-dead voluntary organ donors with no history of back pain. The diseased discs (DD) group had NP tissues of 20 diseased discs excised from 20 patients undergoing spinal surgery as per the ethical norms laid by the Indian Council of Medical Research (ICMR; Table 1).

**Table 1:** Demographic details of the study population.

**Sample ID**	**Study group**	**Sample type**	**Age**	**Sex**	**Disc level**	**Pfirmann grade**
ND001	ND	NP, EP	43	M	L3–L4	2
ND002	ND	NP, EP	22	M	L3–L4	3
ND003	ND	NP, EP	22	M	L4–L5	2
ND004	ND	NP, EP	22	M	L5–S1	2
ND005	ND	NP, EP	55	F	L4–L5	2
ND006	ND	NP, EP	67	M	L4–L5	2
ND007	ND	NP, EP	63	M	L4–L5	2
ND008	ND	NP, EP	28	M	L4–L5	1
ND009	ND	NP, EP	56	F	L3–L4	2
ND010	ND	NP, EP	56	F	L4–L5	2
ND011	ND	NP, EP	56	F	L5–S1	2
ND012	ND	NP, EP	45	M	L4–L5	2
ND013	ND	NP, EP	32	F	L2–L3	2
ND014	ND	NP, EP	32	F	L3–L4	3
ND015	ND	NP, EP	32	F	L4–L5	2
ND016	ND	NP, EP	32	F	L5–S1	3
DD001	DD	NP	38	F	L5–S1	5
DD002	DD	NP	21	M	L5–S1	3
DD003	DD	NP	37	M	L3-L4	3
DD004	DD	NP	43	M	L5–S1	4
DD005	DD	NP	37	M	L4–L5	3
DD006	DD	NP	15	M	L4–L5	1
DD007	DD	NP	38	M	L4–L5	4
DD008	DD	NP	38	M	L4–L5	4
DD009	DD	NP	43	F	L4–L5	4
DD010	DD	NP	43	F	L4–L5	4
DD011	DD	NP	45	F	L4–L5	4
DD012	DD	NP	44	F	L4–L5	4
DD013	DD	NP	16	F	L5–S1	4
DD014	DD	NP	42	M	L3–L4	5
DD015	DD	NP	69	F	L5–S1	4
DD016	DD	NP	60	M	L4–L5	4
DD017	DD	NP	78	M	L2–L3	4
DD018	DD	NP	78	M	L2–L3	4
DD019	DD	NP	69	F	L4–L5	5
DD020	DD	NP	12	M	L5–S1	2

ND, Normal Discs; DD, Diseased Discs; NP, Nucleus Pulposus; EP, End Plates; M, Male; F, Female.

### Ethical clearance and IRB approval

Intervertebral discs from brain-dead organ donors were collected in full agreement with TRANSTAN-214/2016 (Transplant Authority Government of Tamil Nadu, India). Institutional review board (IRB) approval (10/09/2020) for the study was obtained from the IRB of Ganga Medical Center and Hospital, Coimbatore. Ethical clearance for the study was obtained from the ethics committee (NECRBHR Regn No: EC/NEW/INST/2020/1146), and written consent was obtained from all the patients.

### Extraction of genomic DNA

Disc tissues were collected under sterile operating conditions and snap frozen in sterile cryovials using liquid nitrogen (−196°C). DNA was extracted from 200 mg of intervertebral disc tissues—NP and EP tissues of ND group and NP from DD group using QIAamp^®^ DNA Mini Kit—and enrichment of bacterial DNA was performed using NEBNext^®^ Microbiome DNA Enrichment Kit, #E612S/L (New England BioLabs, Ipswich, MA), as per the manufacturer's instructions.

### Quality controls

To check for potential contamination of foreign DNA during PCR amplification, process control [PBS buffer, No template control (NTC)] was substituted for template DNA, and positive control (*E. coli* sample) was used. No spurious DNA amplification was observed for buffers used for processing tissue samples procured from patients for the study (Supplementary Figure 1).

### 16S rRNA amplicon sequencing

The DNA samples were purified, assessed for their quality, and further used for library construction and amplification of 16S rRNA using V1-V9 primers (Table 2). About 25 ng of DNA was used to amplify bacterial 16S rRNA hypervariable region V1–V9 using DNA KAPA HiFi HotStart Ready Mix and 100 nm of the three primer pairs 7F-531R, 513F-1066R, and 1097F-1535R. Amplified fragments were sequenced using the Illumina MiSeq platform as described earlier (4).

**Table 2:** Details of primers used for amplifying the variable regions of 16S rRNA.

**S. No**	**Primers**	**Primer Region**	**Primer Details (5^′^ → 3^′^)**	**Amplicon Size**
1	V13	7F	AGAGTTTGATGMTGGCTCAG	525 bp
2		531R	TTACCGCGGCMGCSGGCAC	
3	V46	513F	GTGCCAGCTGCCGCGGTAATAC	554 bp
4		1066R	CTGACGACAGCCATGCA	
5	V79	1097F	GCAACGAGCGCAACCCC	439 bp
6		1535R	AAGGAGGTGATCCAGGC	

### Bioinformatic analysis

#### Identification of ASVs

Demultiplexed paired-end (2 × 301bp) raw reads were imported into the Quantitative Insights Into Microbial Ecology-2 QIIME2 (v 2020.8) pipeline (9). Further, primers and adaptor sequences were trimmed using the q2-cutadapt plugin. Single-end reads were used for comparing the microbiome colonizing NP and EP of normal IVD, and paired-end reads were used for profiling the microbiome in the NP tissues of ND and DD groups. DADA2 (10) plugin in QIIME2 was used to produce amplicon sequence variants (ASV) feature tables and representative sequences.

#### Normalization of identified ASVs

Taxonomic classification was assigned using the Greengenes database (version 138) integrated within QIIME2 followed by phylogenetic diversity analysis. Unassigned reads and chloroplast (cyanobacteria) and mitochondria reads were also removed from ASVs. ASVs were normalized *via* simple division to their sample size and then multiplied by the size of the smaller sample and low prevalence ASVs (<0.025%) were also filtered from the feature table to strengthen the analysis (11). A rarefaction curve was built at the sampling depth of 16,000, which retained 36.91% (688,000) of sequences in 100% of samples. This sampling depth was selected based on the maximum depth that retained all samples for further analysis.

#### Phylogenetic diversity analysis

**Figure 1:**
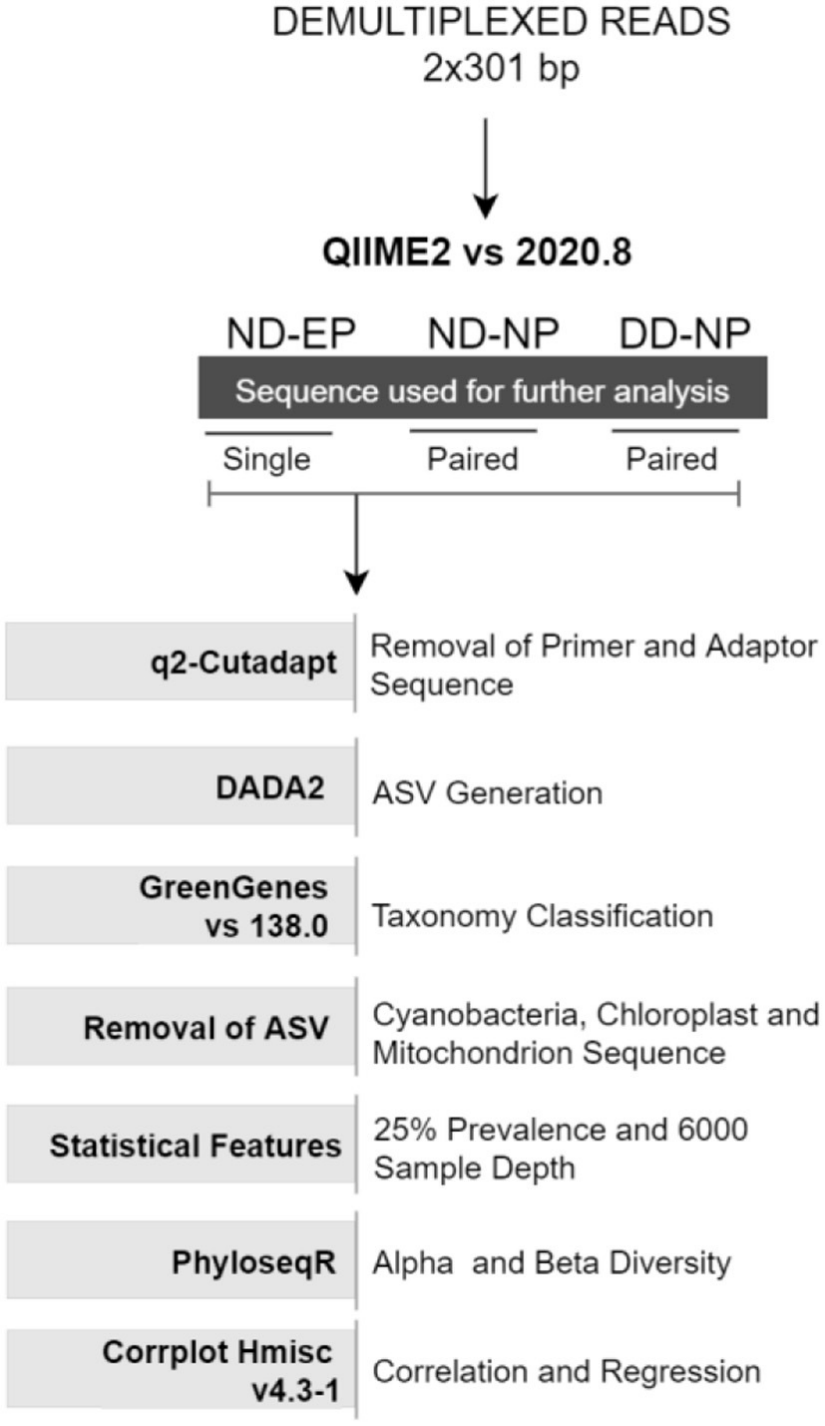
Data analysis workflow deployed for the study.

Alpha diversity indices (Shannon and Observed OTU) and beta diversity analyses were performed using the unifrac weighted and unweighted phyloseq R package (12). Non-metric multidimensional scaling (NMDS) was used to investigate the (dis)similarities between bacterial communities for weighted unifrac methods, as found in Figure 1.

### Correlation and regression analysis

Correlation between changes in the relative abundance of different phyla during health and disease conditions was estimated using Spearman's rank correlation coefficient test using the rcorr function in Hmisc v4.3-1 R package and visualized using the corrplot function in R with a significance level set at 0.05.

### Data availability

Illumina paired metagenomic sequencing data generated for all samples in this study are deposited in the NCBI Sequence Read Archive database (https://www.ncbi.nlm.nih.gov/sra) under BioProject ID PRJNA577202 and PRJNA831905 for NP and EP tissues, respectively.

## Results

This study attempted to analyze the possible route of microbial entry into the NP tissues of IVD by profiling the bacterial population(s) in the EP and NP of the intervertebral disc and to analyze the alterations in the diversity and abundance of bacterial species during health and disease conditions. To the best of our knowledge, this is the first systematic report on the tissue-specific microbial profiling in the NP and EP tissues of the human intervertebral disc (IVD). One of our earlier studies reported a detailed analysis of the microbiome inhabiting nucleus pulposus (NP) tissues of IVD (4). This study documented changes in the diversity and abundance of bacteria between ND (16 NP and 16 EP tissues excised from the brain-dead voluntary organ donors) and DD (20 NP tissues excised from patients undergoing spinal surgery) groups using NGS-supported 16S rRNA amplicon sequencing.

### PCR quality controls

Process controls (PBS and NTC) were used to evaluate contamination from extraneous DNA materials present in reagents or buffers used for metagenome sequencing. Process controls had shown no amplification while amplifying with universal primers whereas positive control showed amplification (Supplementary Figure 1).

### Microbial profile of NP and EP of normal IVD

Microbiome profiling in the NP and EP tissues of normal IVD through 16S rRNA amplicon sequencing generated 141,747 and 136,569 reads, respectively. The quality of raw reads was relatively comparable between the tissues (Supplementary Table 1). Raw reads were subjected to the removal of primer and adaptor sequences using the DADA2 denoising algorithm and the obtained ASVs were mapped against Greengenes vs. 138 database for assigning OTUs. Nearly 99.5% of the reads were classified at the kingdom level, 51.7% of sequences were classified at the phylum level, 21.9% of the reads were assigned at the genus level, and <1% of reads remained uncharacterized.

**Figure 2:**
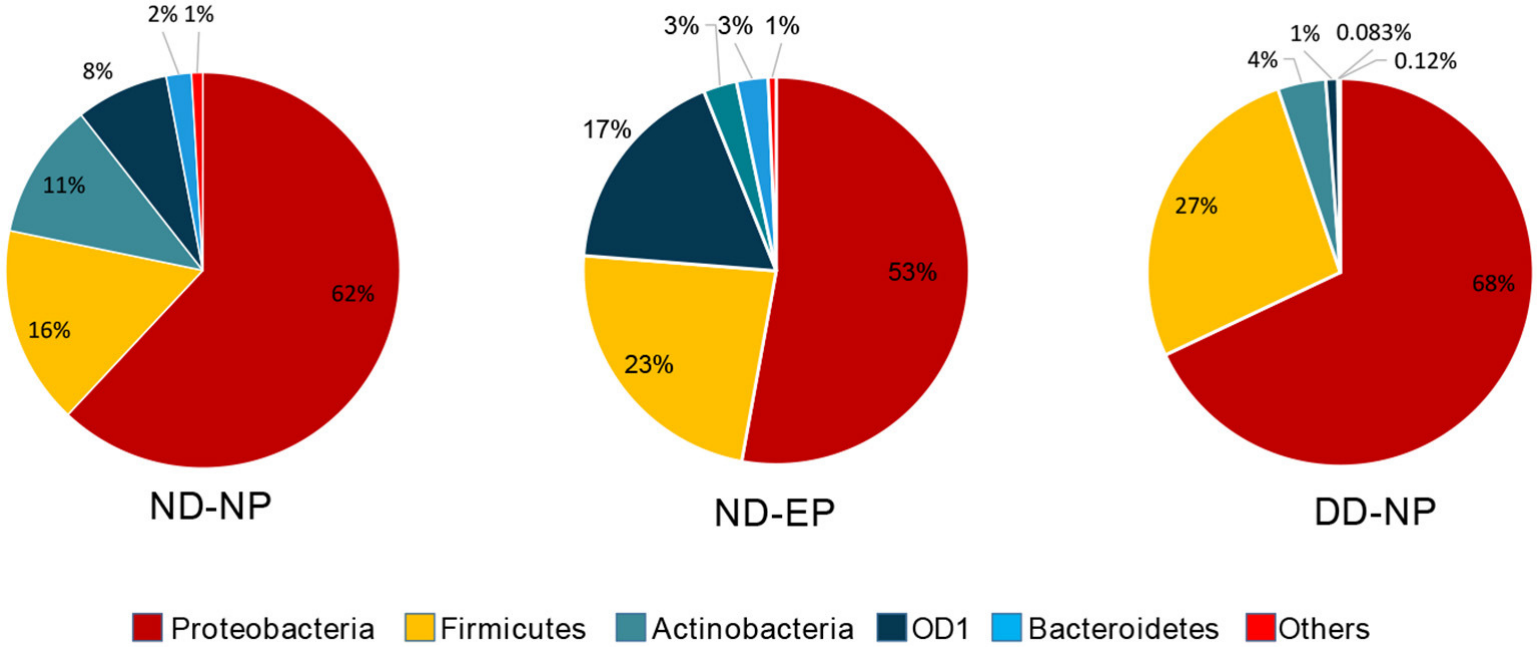
Abundance at Phylum level. Donut plots represent the relative abundance of phylum across ND (NP, EP) and DD (NP) groups. *Proteobacteria* was the most abundant phyla compared to other phyla.

At the Phylum level, five different phyla, namely, *Proteobacteria, Firmicutes, Actinobacteriota, OD1 (Parcubacteria)*, and *Bacteroidetes*, were present with >1% relative abundance. *Proteobacteria* remained as the most abundant phylum in both NP (62%) and EP (53%) tissues of normal IVD. This was followed by *Firmicutes (16%), Actinobacteriota (11%), OD1 (Parcubacteria) (7.6%), and Bacteroidetes (2%) in NP and Firmicutes (23.4%), OD1 (Parcubacteria) (17.6%), Actinobacteriota (2.8%), and Bacteroidetes (2.6%) in EP, respectively* (Figure 2). It was interesting to note that the top five phyla were similar between NP and EP.

**Figure 3:**
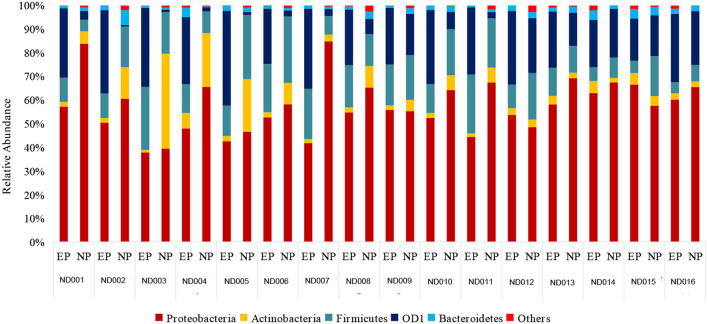
Comparison of bacterial phyla in NP and their respective EP of normal discs. *Proteobacteria* and *Firmicutes* were slightly abundant in NP when compared to EP.

Essentially, a *t*-test was calculated to find the significance between the NP and EP. As the study was performed based on QCA (Qualitative Comparative Analysis), FDR-control procedures were calculated to signify incorrect rejection from the comparison between NP and EP. We found an FDR <0.01 in the QCA of *Proteobacteria* (*p* < 0.001), *Actinobacteria* (*p* < 0.015), *Firmicutes* (*p* < 0.03) and *OD1* (*p* < 0.001) in the calculated parametric test. *Proteobacteria, Actinobacteria* were significantly decreased whereas *Firmicutes* and *OD1* were significantly increased in EP comparatively (Figure 3).

**Figure 4:**
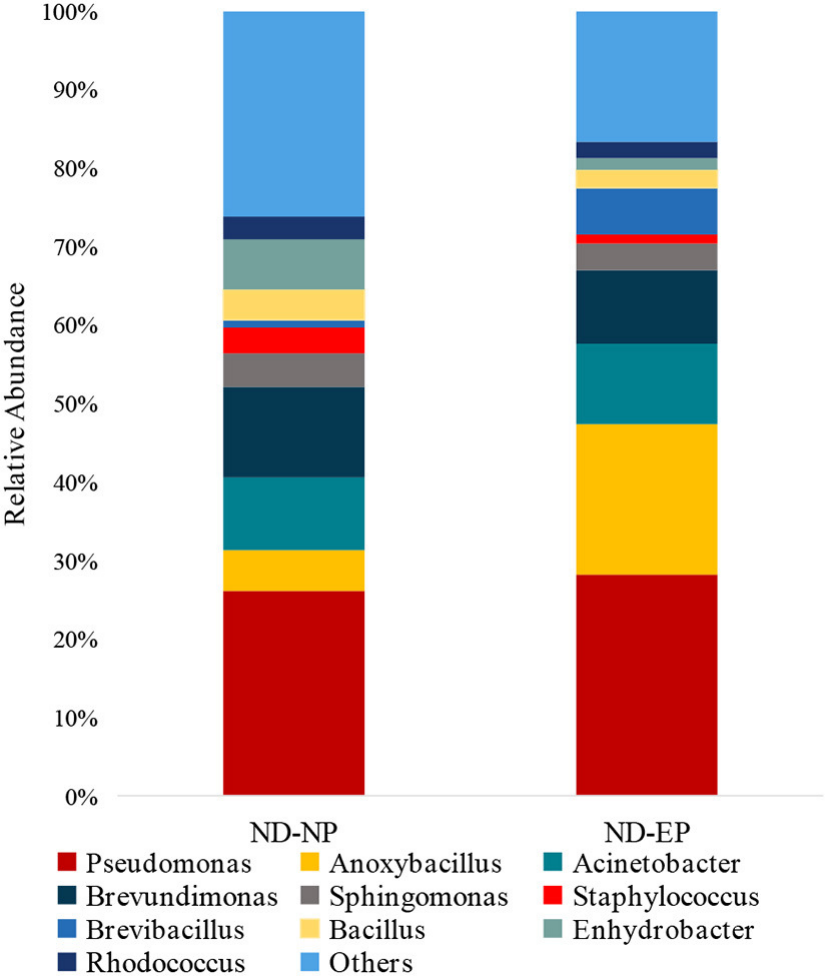
Abundance of microbiome at Genus level (ND-NP vs. ND-EP). Stacked bar plots representing the relative abundance of phylum across ND-NP and ND-EP groups. *Pseudomonas* belonging to the phyla *Proteobacteria* were found to be dominant with higher abundance in both ND-NP (26%) and ND-EP (28%).

The relative abundance of the top 15 genera across NP and EP tissues of normal IVD is depicted in Figure 4. *Pseudomonas* belonging to the phyla *Proteobacteria* were dominant with higher abundance in both ND-NP (26%) and ND-EP (28%) compared to all other identified genera. In NP discs, the second most abundant genus was *Brevundimonas* (12%) which was relatively lower in EP discs (9%). In contrast, EP discs had *Anoxybacillus* (19%) to be the second most abundant genus when compared to NP (5%). To validate the NGS results, qPCR analysis was performed for the abundant bacterial genus in five representative EP and NP components of the human intervertebral disc tissue. As *Pseudomonas* is the abundant genus identified in our 16S NGS data, we used the *Pseudomonas* genus-specific primer for amplification using SYBR green-based chemistry on Bio-Rad CFX Opus 96 Real-Time PCR system. A Cq value for each sample was determined (Cq range 26.05–29.57), indicating the abundance of *Pseudomonas* in all sample types (Supplementary Data).

### Diversity of bacterial communities across EP and NP (ND)

In comparing the relative abundance of the bacterial phyla between NP and EP discs of normal donors, we found that *Proteobacteria* and *Actinobacteria* were slightly decreased in EP compared to NP, whereas an enriched microbial abundance of *OD1 and Firmicutes* in EP was found (Figure 3).

**Figure 5:**
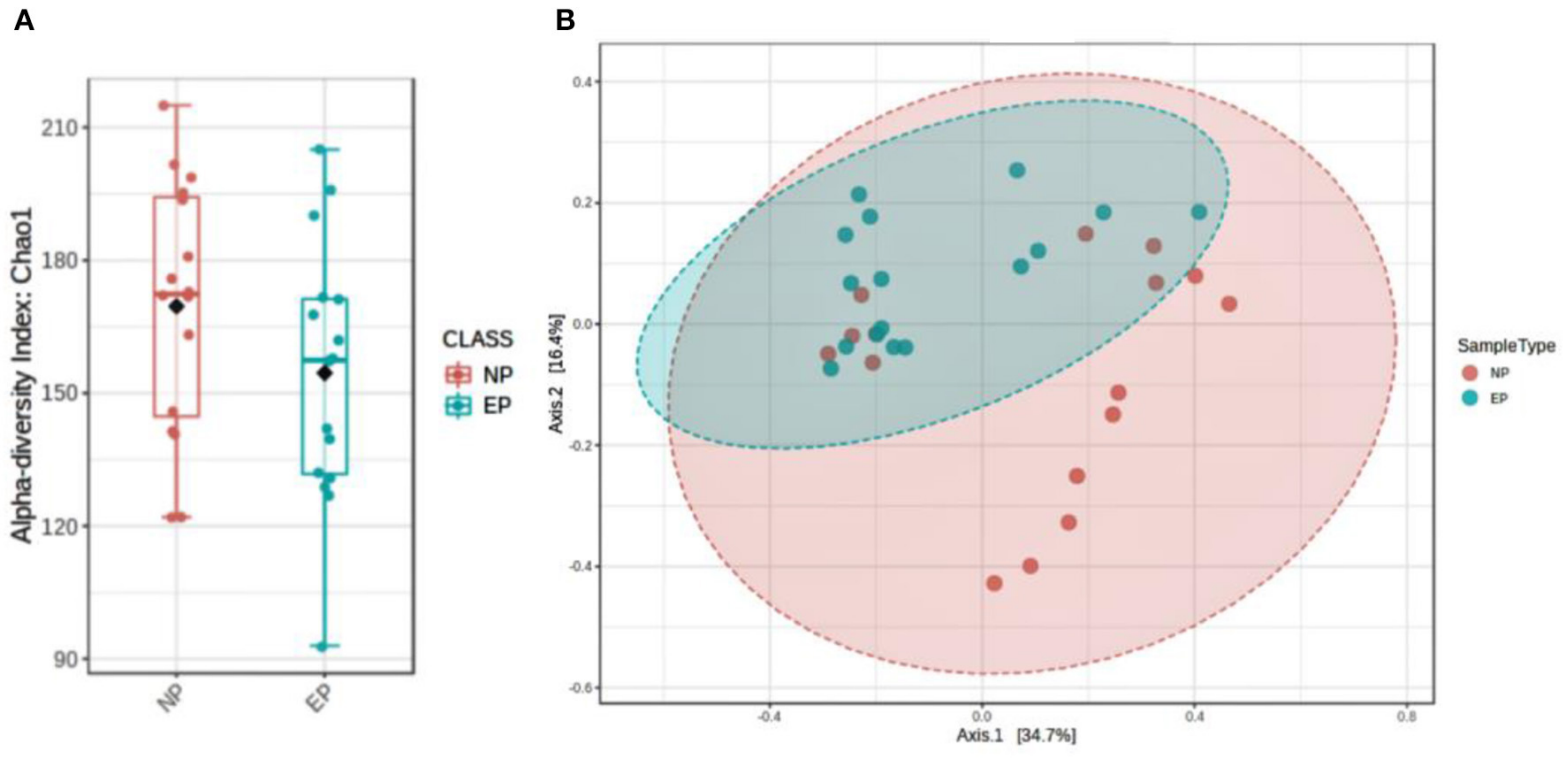
Assessment of microbial diversity. **(A)** Two indices depict alpha diversity: observed OTUs and Shannon indices showing insignificant differences tested using a non-parametric test and the Wilcoxon rank-sum test. **(B)** Beta diversity calculated using phylogenetic methods (UniFrac distance) shows a significant difference in the weighted (p, 0.006) index.

To assess the diversity of bacteria colonizing NP and EP tissues of ND, alpha diversity was determined through estimating observed OTUs and Shannon indices (Figure 5A), and significance was tested using a non-parametric test and the Wilcoxon rank-sum test. NP and EP tissues did not differ much in terms of their observed number of OTUs (*p* = 0.15) and Shannon index (*p* = 0.8). Beta diversity estimates, that is, weighted UniFrac distance, indicated the significant difference between the EP and NP tissues (*p* = 0.006) (Figure 5B).

### The alteration of NP microbiome during disease

There was a shift in the abundance of dominant bacteria colonizing NP tissues of IVD in degenerated discs. The dominant phyla *Proteobacteria* remained at the top in the NP tissues of both ND and DD (67.99% in DD as against 62% in the ND), as shown in Figure 2. This was followed by *OD1 (Parcubacteria) (26.7%), Firmicutes (4.02%), Actinobacteriota (1.0%), and Bacteroidetes (0.08%). OD1 (Parcubacteria)* showed a significant increase (7.6% in ND to 26.7% in DD) during disease progression. The other three Phyla, *Firmicutes* (16% to 4%), *Actinobacteria* (11–1%), and *Bacteroidetes* (2–0.08%), showed several-fold reduction during disease.

**Figure 6:**
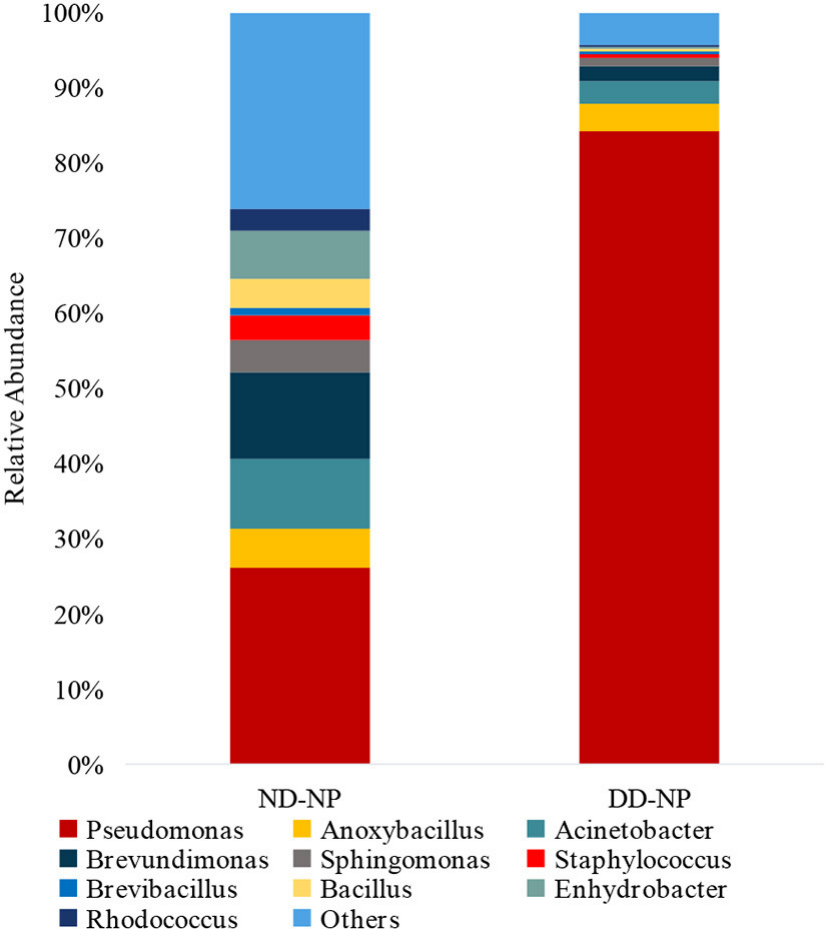
Abundance of microbiome at Genus level (ND vs. DD). Stacked bar plots representing the relative abundance of phylum across ND-NP and DD-NP groups. *Pseudomonas* was the most abundant genera (84%) compared to all other identified genera.

In comparing bacterial genera between ND-NP and DD-NP tissues, *Pseudomonas* was the most abundant genus in DD-NP (84%) and is strikingly higher than ND-NP (26%) as shown in Figure 6. In contrast, other genera, including *Brevundimonas, Anoxybacillus, and Bacillus*, were strikingly lesser in DD than ND.

### Altered ratio of A+F and proteobacteria during DD

**Figure 7:**
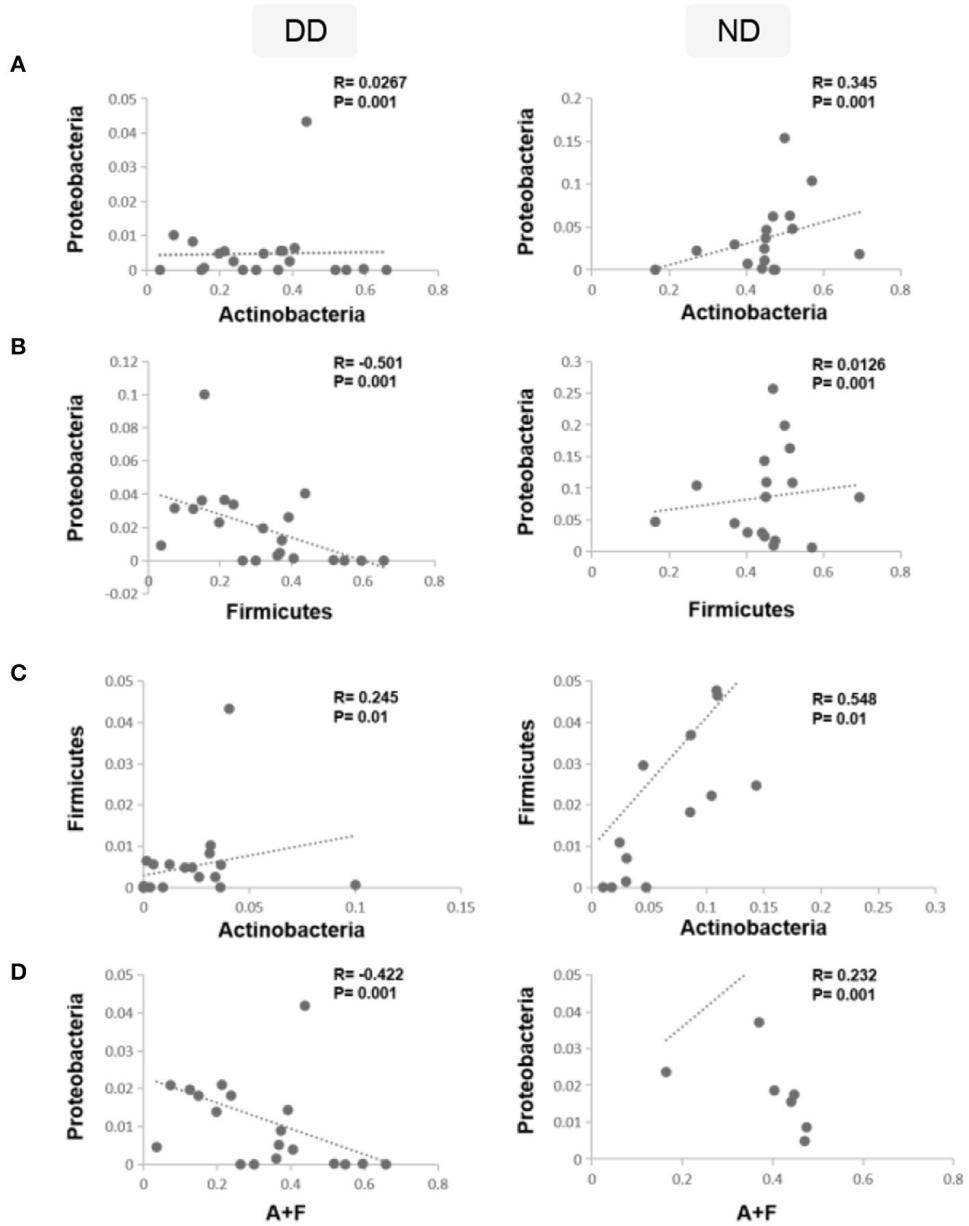
Correlations between dominant phyla and disease condition. Spearman's rank correlation coefficient was used to analyze the correlation between dominant phyla, ND, and DD. **(A)** The relative abundance of *Actinobacteria* and *Proteobacteria* was correlated positively in ND and found significant, whereas, in DD, it demonstrated a weak positive correlation. **(B)** *Firmicutes* and *Proteobacteria* showed a weak positive correlation in ND, whereas DD demonstrated a significant negative correlation. **(C)** *Actinobacteria* and *Firmicutes* exhibited a positive correlation and were found significant in both ND and DD. **(D)** Ratio of *Actinobacteria* + *Firmicutes: Proteobacteria* showed a significant negative correlation during DD and a significant positive correlation in ND.

To analyze the shift in the abundance of bacteria during disease (DD), Spearman correlation analysis was carried out using the rcorr function in the Hmisc R package. The correlation analysis revealed that the relative abundance of *Actinobacteria* and *Proteobacteria* exhibited a weak positive correlation (*p* = 0.001) in DD, whereas in ND, it demonstrated a moderate positive correlation (*R* = 0.345; *p* = 0.001) (Figure 7A). *Firmicutes* and *Proteobacteria* showed a significant negative correlation (*R* = −0.501) in DD, whereas, in ND, a weak positive correlation was observed (Figure 7B). *Actinobacteria* and *Firmicutes* exhibited a positive correlation and were found significant in both ND (Figure 7C). The ratio of *Actinobacteria* + *Firmicutes*: *Proteobacteria* showed a significant negative correlation during DD (*R* = −0.422). In contrast, ND showed a moderate positive correlation (*R* = 0.232) which was significant (Figure 7D).

## Discussion

Traditionally intervertebral discs with intact annulus fibrosus were considered avascular (8). Hence, the reports of bacterial presence in intervertebral disc specimens obtained during surgery were met with criticism and deemed as contamination (13). However, growing evidence documents the bacterial presence in the IVD using various modalities (3, 4, 14). Our earlier study had proved the existence of a “Microbiome” within the intervertebral disc in both normal discs and degenerated discs using NGS techniques and led to the questions of how and when bacteria colonized the disc, what is their route of entry into IVD, and their role in health and disease (4).

The nutrition supply to NP tissue occurs *via* chemical diffusion through the capillaries that arise in vertebrate bodies and traverse to the cartilaginous endplates (EP) (15). The rate of nutrient transport is determined by the rate of cellular demand and the concentrations of nutrients, oxygen (< 5%), and pH (< 6.0) at the center of the discs comprising NP (16). We hypothesized that the endplate would not only be a gateway for nutrition but also the microbiome.

### Bacterial presence vs. contamination

The bacterial presence in the intervertebral disc has been criticized as contamination in view of its avascular nature. However, there is growing evidence to the contrary. Recent studies have documented the existence of a microbiome within IVD (4). In our study, we procured the disc tissues in a sterile operating room and have Snap-froze them immediately to avoid contamination. In the lab, the samples were handled under sterile precautions for DNA extraction. We utilized positive and negative controls for PCR amplification and our results verified that there was no contamination during the experiment. Using metabolomics, a recent study has documented the presence of bacterial metabolites in both control and degenerated discs indicating that the bacteria in discs are not only alive but are multiplying and active metabolically ruling out the possibility of contamination (17).

### Nucleus pulposus and endplate share highly similar microbiome

Our results showed that the microbiome inhabiting EP and NP tissues of normal discs were almost identical. *Proteobacteria* was the predominant phyla in both tissues, followed by *Firmicutes*. There were only minor differences between the two tissues in the abundance of *Actinobacteria* and OD1, which resulted in slight changes in the order of abundance of the top five phyla of microflora inhabiting IVD. Nevertheless, the overall bacterial composition was identical in EP and NP.

At the genus level*, Pseudomonas was the* dominant genus in both EP and NP. *Pseudomonas* is a gram-negative aerobe and has the ability to survive under adverse conditions. Although it is well known for its pathogenicity, it can degrade a large number of toxic compounds. It is also known for its industrial uses and beneficial effect on plants as it represses many peripheral catabolic enzymes and helps in biocontrol (18, 19). Their role in human IVD needs to be investigated. The next abundant genus was *Anoxybacillus*, a heat-tolerant bacteria that have been shown to have a role in human cellular metabolism and protein folding.

In addition, Anoxybacillus has been used commercially to produce maltose in the baking industry and in bioremediation to remove toxic metals and as a probiotic and stimulant of the immune system in fish against pathogens (20). *Brevundimonas* are found in abundance in normal NP, and they are carotenoid-producing bacteria. These carotenoids are potent human antioxidants that play an essential role in preventing tissue damage from occurring secondary to immune and inflammatory activity. The source of these antioxidants could be either dietary or bacterial (21). Hence, these antioxidants could play a role in maintaining extracellular matrix (ECM) integrity and tissue homeostasis (22).

### Functional diversity of microbiome inhabiting NP and EP

To analyze the overall differences in the bacterial composition, we performed alpha diversity analysis using observed OTUs and Shannon indices. Alpha diversity represents the mean species diversity in sites or habitats. It was not surprising that there was no significant difference between NP and EP in terms of their microbial composition, which adds strength to our hypothesis that microbial colonies enter NP through EP. The minor differences in their metagenomic profile between NP and EP could have arisen as a result of the difference in their microenvironment, that is, vascularity, oxygenation, and pH between the two (23, 24).

### Origin of microbiome

Although human IVD is avascular, endplate breaks resulting from microtrauma occur as early as the second decade and lead to disc-marrow contact (25). Although our study proves that endplate could be the route of entry for microbes into NP, the origin of the microbiome remains speculation. The majority of bacteria found in our study are common inhabitants of the human gut, leading us to believe that many gut bacteria *via* the hematogenous route could have been harbored in NP. However, this needs to be proven by a study comparing the disc and gut microbiomes of the same individuals.

### Changes in the abundance of microflora between health and disease

To understand the alterations in microbial diversity in DDD, we compared the microbiome of normal discs, which showed a significant decrease in *Firmicutes* and *Actinobacteria*. They form two of the most abundant phyla in the human gut, and their decreased abundance in the human gut has been associated with dysbiosis and many inflammatory diseases (26). In addition, *Vaiserman* et al. reported the altered ratio of relative abundance of the top abundant bacteria, *Firmicutes: Bacteroidetes*, in the gut as a biomarker determining health and disease (27).

Similarly, the correlation between dominant phyla of health and disease conditions was studied. While Firmicutes and Proteobacteria showed a positive correlation in ND, they demonstrated a significant negative correlation in DD. This led us to investigate whether the ratios of the abundant phyla observed in ND are altered in DD. While evaluating the ratios of various phyla across ND and DD, the ratio of *Firmicutes/Proteobacteria* showed a significant negative correlation in DD compared to *Actinobacteria/Proteobacteria* (positive correlation) and *Actinobacteria/Firmicutes* (positive correlation). Interestingly, the combined ratio of *Actinobacteria* + *Firmicutes/Proteobacteria* showed a negative correlation that may be of potential significance in the progression of DD.

Bacteria are present in all areas of the human body as commensals in every individual and play a vital role in gut metabolism, immunity, and so on (28). Approximately, 98% of the microbiome of blood is primarily comprised of Proteobacteria followed by Bacteriodetes, Actinobacteria, and Firmicutes, with a higher abundance of Proteobacteria in patients with chronic kidney disease (CKD) (29). Proteobacteria encompasses a wide spectrum of bacteria which may be either commensal, pathogenic, or opportunistic pathogens (depending upon host factors, they may become pathogenic) (30). However, the hematogenous spread of bacteria from one location to other is well known and does not always result in sepsis (31). The bacterial seeding that occurs is very minimal and unless there is a suitable environment and favorable host factors, they do not result in overt infection.

### Clinical significance

The last decade has witnessed a change in our understanding of human bacterial interaction and the role of bacteria in health and disease (32). Many diseases that were thought to be idiopathic or inflammatory have been proven to have a microbial origin or have a close association (33). Recent studies have also focussed on altering the gut microbiota by the use of probiotics to restore health and induce remission in many chronic illnesses (34–36). The findings of our study prove the existence of a “microbiome” and the possible route of entry into NP is *via* EP, and “dysbiosis” plays a possible role in disc degeneration. Our earlier study had proven that the diffusion across the endplates could be altered by drugs, such as Nimodipine (2, 37). In this context, altering the gut microbiome in restoring IVD health and its facilitation by pharmacological enhancement remains a possible avenue to be explored.

### Limitations and future applications

In NGS metagenome data of EP, we used single-end reads for our analysis due to the quality of reads. The ratio of *Actinobacteria* + *Firmicutes: Proteobacteria* is of significance in DD; this finding needs to be validated in a larger number of samples. It would be interesting to employ a multi-omic approach comprising metabolomics, metaproteome, and host proteome to understand the intricate crosstalk between the host and microbial colony and its effect on disc health. Further analyzing the fetal IVD microbiome will throw further evidence to the above findings.

## Conclusion

Our metagenome study has demonstrated a close similarity in the metagenomic profile of human lumbar intervertebral disc nucleus pulposus and the endplate, suggesting a possible existence of the endplate–nucleus pulposus axis in normal IVD. Minor variations between the microbiome in normal NP and EP tissues reiterate the sterility of disc, where the shift in their proportional existence could alter the health status. The increased abundance of *Proteobacteria* with a significant reduction in *Firmicutes, Actinobacteria*, and *Bacteroidetes* could be a potential microbial signature in the progression of DD. In addition, the altered ratio of dominant phyla *Actinobacteria* + *Firmicutes: Proteobacteria* during diseased states needs further investigation.

## Data availability statement

The datasets presented in this study can be found in online repositories. The names of the repository/repositories and accession number(s) can be found below: BioSample, PRJNA577202 and PRJNA831905.

## Ethics statement

The studies involving human participants were reviewed and approved by the Institutional Review Board of Ganga Medical Center and Hospital, Coimbatore (No: 2020/09/10). Written informed consent to participate in this study was provided by the participants' legal guardian/next of kin.

## Author contributions

SR, CT, RM, and SKS were involved in conceptualization and supervision of the project. CT and SN designed and performed the experiments. MM, SN, and RS did the extensive data analysis. SN and MM wrote the first draft of the article which was further improvised by SR, CT, RM, and SKS. SR, CT, RM, SKS, RK, and AS involved in review and editing. All authors contributed to the article and approved the submitted version.

## Funding

The study was funded by Ganga Orthopaedic Research and Education Foundation (GOREF) (GOREF-2019/12) (Recognized by Department of Scientific and Industrial Research (DSIR) and co-funded by Department of Biotechnology (DBT), Government of India, Grant reference no: BT/PR35631/MED/30/2186/2019.

## Conflict of interest

The authors declare that the research was conducted in the absence of any commercial or financial relationships that could be construed as a potential conflict of interest.

## Publisher's note

All claims expressed in this article are solely those of the authors and do not necessarily represent those of their affiliated organizations, or those of the publisher, the editors and the reviewers. Any product that may be evaluated in this article, or claim that may be made by its manufacturer, is not guaranteed or endorsed by the publisher.
