# Supplementary material for: Comparative metagenomic analysis of human intervertebral disc nucleus pulposus and cartilaginous end plates
Source: Front Cardiovasc Med. 2022 Sep 28;9:927652. doi: 10.3389/fcvm.2022.927652 (PMC9554234; doi:10.3389/fcvm.2022.927652)
Supplement: Supplementary file 1 [file Data_Sheet_1.docx]

**Supplementary Figure-1: Quality check of process controls for monitoring extraneous DNA contamination.**

**Supplementary Table-1: Mean quality scores of raw reads of NP and EP.**

| **Description** | **NP** | | **EP** | |
| --- | --- | --- | --- | --- |
| **Sample ID** | **Q20** | **Q30** | **Q20** | **Q30** |
| ND001 | 85.2 | 72.4 | 73.8 | 61.6 |
| ND002 | 87.9 | 75.1 | 76.8 | 65.3 |
| ND003 | 86.7 | 73.2 | 75.1 | 63.4 |
| ND004 | 86.5 | 73.5 | 75.0 | 63.0 |
| ND005 | 88.8 | 75.8 | 75.4 | 63.5 |
| ND006 | 85.4 | 72.7 | 75.7 | 63.9 |
| ND007 | 83.1 | 69.7 | 75.7 | 63.7 |
| ND008 | 81.8 | 68.9 | 74.4 | 62.5 |
| ND009 | 78.9 | 66.5 | 74.4 | 62.4 |
| ND010 | 77.0 | 64.0 | 73.5 | 61.8 |
| ND011 | 84.5 | 71.8 | 75.2 | 63.3 |
| ND012 | 74.2 | 62.2 | 73.6 | 60.8 |
| ND013 | 75.5 | 63.5 | 82.8 | 70.6 |
| ND014 | 75.7 | 63.6 | 83.3 | 69.1 |
| ND015 | 74.9 | 62.9 | 82.4 | 68.0 |
| ND016 | 74.2 | 62.2 | 82.4 | 67.9 |
| **Mean quality score** | **81.27** | **68.63** | **76.84** | **64.43** |

**Supplementary Data**

**qPCR analysis to validate NGS results using genus-specific primers:**

To validate the NGS results of abundant bacteria inside intervertebral disc tissue qPCR analysis was carried out.

**Methodology:**

Bacterial DNA was extracted from five representative NP and EP intervertebral disc tissue samples post depletion of host DNA using Qiagen QIAmp® DNA microbiome Kit (#Cat. No. 51704). Extracted bacterial DNA’s quality and quantity was measured at A_260_/A_280_ ratio using nanodrop Varioskan Lux spectrophotometer. The range was between 1.8 - 2.0, and all samples were subjected to DNA amplification using genus specific primer for the abundant genus *Pseudomonas*.

*Pseudomonas*, being the abundant Genus identified in our 16S NGS data, we used the *Pseudomonas* genus specific primer for amplification using SYBR green based chemistry on Bio-Rad CFX Opus 96 Real-Time PCR system as per the set conditions found in below table-1.

**Table 1**: PCR protocol for genus specific amplification.

| **Assay** | **Primers** | **Thermal Conditions** | **No. of Cycles** |
| --- | --- | --- | --- |
| rpoD | Forward - ATYGAAATCGCCAARCG  Reverse - CGGTTGATKTCCTTGA | 98ºC for 3 minutes | 1 |
|  |  | 95ºC for 15 seconds | 35 |
|  |  | 51ºC for 30 seconds |  |
|  |  | 72ºC for 20 seconds |  |
|  |  | **Melt curve analysis**  57ºC - 95ºC for 5 seconds | 0.5ºC/ cycle |

**Genus specific amplification:**

Representative five NP and EP samples were used for amplification and two no template control (NTC) were used (water and 1X PBS buffer were substituted for template DNA). A Cq value was obtained for each of the sample subjected to amplification, no amplification could be observed in NTC. The results of NGS was well correlated with qPCR amplification (**Table 2 and Figure 1**).

**Table 2**: qPCR analysis for *Pseudomonas* genus specific primer using SYBR green chemistry. Cq values represent the abundance of *Pseudomonas* genus in each sample.

| **Well** | **Fluor** | **Target** | **Content** | **Sample ID** | **Cq value** |
| --- | --- | --- | --- | --- | --- |
| A01 | SYBR | rpoD | NTC | Water | NaN |
| A02 | SYBR | rpoD | Unkn | ND006/NP | 26.71 |
| B01 | SYBR | rpoD | Unkn | ND014/EP | 29.57 |
| B02 | SYBR | rpoD | Unkn | ND009/EP | 26.05 |
| C01 | SYBR | rpoD | Unkn | ND014/NP | 26.40 |
| C02 | SYBR | rpoD | Unkn | ND009/NP | 26.05 |
| D01 | SYBR | rpoD | Unkn | ND007/EP | 26.69 |
| D02 | SYBR | rpoD | NTC | PBS | NaN |
| E01 | SYBR | rpoD | Unkn | ND007/NP | 26.08 |
| F01 | SYBR | rpoD | Unkn | ND012/EP | 26.16 |
| G01 | SYBR | rpoD | Unkn | ND012/NP | 27.94 |
| H01 | SYBR | rpoD | Unkn | ND006/EP | 28.00 |

Unkn- Unknown sample ; NTC- No Template Control


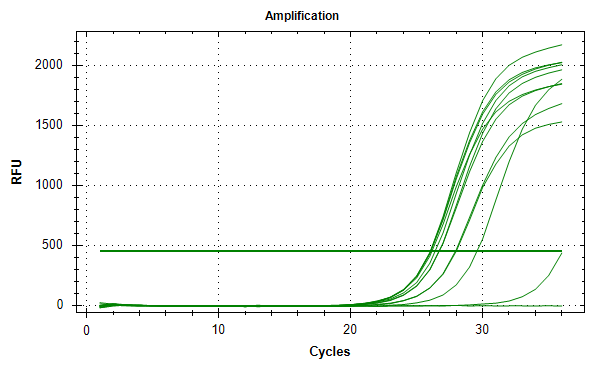


**Figure 1:** Real-Time PCR amplification plot depicted for 10 ng of DNA of IVD NP and EP tissues. The chart was generated by plotting relative fluorescence (RFU) vs. cycle number. NTC is referred as no template control.
